# Respiratory support strategies in the prevention and treatment of bronchopulmonary dysplasia

**DOI:** 10.3389/fped.2023.1087857

**Published:** 2023-03-01

**Authors:** Erik B. Hysinger, Shawn K. Ahlfeld

**Affiliations:** ^1^ Division of Pulmonary Medicine, Department of Pediatrics, University of Cincinnati College of Medicine, Cincinnati Children’s Hospital Medical Center, Cincinnati, OH, United States; ^2^ Division of Neonatology, Department of Pediatrics, University of Cincinnati College of Medicine, Cincinnati Children’s Hospital Medical Center, Cincinnati, OH, United States

**Keywords:** prematurity, bronchopulmonary dysplasia, CPAP, mechanical ventilation, tracheostomy

## Abstract

Neonates who are born preterm frequently have inadequate lung development to support independent breathing and will need respiratory support. The underdeveloped lung is also particularly susceptible to lung injury, especially during the first weeks of life. Consequently, respiratory support strategies in the early stages of premature lung disease focus on minimizing alveolar damage. As infants grow and lung disease progresses, it becomes necessary to shift respiratory support to a strategy targeting the often severe pulmonary heterogeneity and obstructive respiratory physiology. With appropriate management, time, and growth, even those children with the most extreme prematurity and severe lung disease can be expected to wean from respiratory support.

## Introduction

Neonates who are born preterm frequently lack adequate lung development to support breathing independently and are quite prone to needing respiratory support, with increasing risk as gestational age decreases. Even during the first days and weeks of life, there are extensive changes in the respiratory system and breathing mechanics for children who are born prematurely, and ventilatory strategies must be titrated to meet each patient's evolving needs and avoid complications. Early in the disease course respiratory support should focus on limiting additional lung injury by utilizing non-invasive ventilation or “gentle” invasive mechanical ventilation with a low tidal volume, short inspiratory time, and high respiratory rate strategy. However, for those infants who go on to need chronic respiratory support, worsening obstructive lung disease requires a transition to longer inspiratory times, lower rates, and a higher tidal volume strategy to optimize ventilation. This review will describe the changes in respiratory support throughout the evolution of premature lung disease.

## Non-invasive ventilation

Although an in-depth discussion is beyond the scope of this review [which is primarily focused on invasive mechanical ventilation of infants at-risk for and with severe bronchopulmonary dysplasia (BPD)], non-invasive ventilation used as both initial support for infants with respiratory distress syndrome and/or as post-extubation support can limit lung injury, minimize exposure to mechanical ventilation, and theoretically can reduce the risk of severe BPD. Excellent, contemporary reviews have thoroughly summarized the safety and efficacy of non-invasive modes of respiratory support (1, 2) including nasal high frequency ventilation (nHFV) (3, 4), non-invasive neurally-adjusted ventilatory assist (NI-NAVA) (5), nasal intermittent positive pressure ventilation (NIPPV) (6), and nasal continuous positive pressure ventilation (nCPAP) (7, 8). Indeed, following publication of the COIN trial in 2008 [the first multicentered, randomized controlled trial demonstrating the safety and efficacy of nCPAP as primary respiratory support for extreme preterm infants with respiratory distress syndrome (9)], from 2008 to 2018 in the United States there has been a shift toward decreased use (and duration) of mechanical ventilation and a concomitant increased use (and duration) of non-invasive ventilation (10). During the same era, however, the incidence of BPD, including severe BPD, has not improved significantly (11–13). Though supported by sound biological plausibility, meta-analysis of the major trials that relied on nCPAP to avoid mechanical ventilation resulted in only a modest (∼10%) reduction in BPD (14). Especially true for infants born at the earliest gestational ages (15), despite efforts to provide non-invasive respiratory support, many infants ultimately require mechanical ventilation. For infants born at 22–28 weeks' gestation and cared for in the Neonatal Research Network between 2013 and 2018, 85% of infants required mechanical ventilation at some point in their hospitalization, and 8% of infants at 36 weeks' corrected gestational age were still ventilator-dependent (16). In the major multicenter randomized controlled trials (RCTs) comparing CPAP to intubation for prophylactic surfactant, by 5–7 days of age ∼50% of extremely preterm infants experienced CPAP failure (9, 17–19). Though reduced by 50% in the recent OPTIMIST-A trial, despite avoidance of endotracheal intubation for minimally-invasive surfactant therapy in the first 6 h of age, by 72 h of age nearly 40% of MIST infants ultimately required intubation and mechanical ventilation (20). Emerging experience with synchronized NIPPV, specifically the use of NIV-NAVA, may prove most beneficial in terms of avoiding initial or subsequent need for mechanical ventilation and hold promise. However, limited options for providing synchronization have hindered wide-spread use. In summary, although questions remain concerning the impact of synchronized noninvasive positive pressure ventilation, despite now over a decade of coordinated efforts to avoid mechanical ventilation, for the majority of extremely preterm infants invasive mechanical ventilation inevitably will be required.

While the majority of infants require a period of invasive mechanical ventilation, by 36 weeks' corrected gestational age, more than 90% will have been extubated and supported non-invasively (16, 21). As stated previously, evidence comparing the efficacy of various modes of non-invasive support is emerging, but presently nCPAP comprises the bulk of available data. Since 2008, 5 large multicenter RCTs (COIN (9), SUPPORT (17), CURPAP (19), Vermont Oxford (18), and now OPTIMIST-A (20)) have enrolled over 3,000 infants born at 24–29 weeks' gestation and cared for with nCPAP and, therefore, provide a wealth of safety and efficacy data. However, despite wide-spread acceptance and use of non-invasive ventilation in both the United States (10) and United Kindom (22), nearly 50% of surviving infants continue to develop BPD (16, 21). Preclinical and clinical evidence implies that outcomes may be improved by prolonging the duration of constant distending pressure. A strategy employing prolonged, prophylactic support on nCPAP until respiratory stability is achieved and infants can be weaned directly to room air is associated with the lowest rates of BPD (23).

Supporting evidence derived from preclinical animal models demonstrates that constant distending pressure minimizes lung injury and augments lung growth. In both murine and rabbit models of hyperoxic neonatal lung injury, compared to no support, use of CPAP reduced inflammation, preserved alveolar-capillary development, and durably improved lung function (24, 25). Exposure of juvenile ferrets to 2 weeks of constant distending pressure significantly increased lung weight and DNA content and increased total lung capacity by 40% while preserving elastic recoil, thus implying CPAP induced not merely lung distension but lung growth (26). In infants with severe congenital diaphragmatic hernia, tracheal occlusion (resulting in lung fluid retention and constant distension of the developing lungs) improved survival and reduced the need for ECMO, strongly-implying improved lung function (27).

Recent clinical evidence indicates that extremely preterm infants with evolving BPD may similarly benefit from prolonged constant distending pressure. Forty-four infants born ≤32 weeks' gestation and requiring ≥24 h of bubble CPAP that had reached clinical stability (i.e., CPAP ≤5 cm H_2_O, fiO_2_ 21%, RR < 70, comfortable work of breathing, minimal cardiorespiratory events, and stability off CPAP for routine care) were randomized to wean directly to room air or remain on bubble CPAP for an additional 2 weeks (28). Infants were randomized at a mean of 32 weeks' corrected gestational age, and extended CPAP was well-tolerated. Despite being similar at randomization, at the end of the treatment period, infants who remained on bubble CPAP for two weeks rather than weaning immediately to room air had significantly larger (∼10%) functional residual capacity (FRC), and the change in FRC was nearly double (12.6 vs. 6.4 ml). At discharge (an average of 2 weeks following discontinuation of the intervention) infants randomized to prophylactic, prolonged nCPAP continued to have a larger FRC (∼20%) that had grown nearly 60% more (27.2 vs. 17.1 ml). Importantly, infants in both groups reached full oral feeds and were discharged at similar corrected gestational ages indicating that extending nCPAP did not prolong hospitalization. A follow-up study will determine the durability of benefits to lung function, but the preliminary results in combination with preclinical data and sound biological plausibility provide promise that prophylactic, prolonged nCPAP may help support and preserve lung development and function.

## Invasive mechanical ventilation

When the severity of respiratory failure requires invasive ventilation, maintaining optimal FRC with appropriate positive end expiratory pressure (PEEP) and avoiding volutrauma through volume targeted ventilation can minimize lung injury. Mechanisms of neonatal lung injury are multifactorial, but include a combination of biotrauma, atelectotrauma, and volutrauma (the concepts for which have been nicely reviewed) (29). Whether acquired prenatally (chorioamnionitis), through vertical transmission of vaginal organisms, or postnatally through ventilator-associated pneumonia, the inflammatory cytokine milieu invoked by neonatal pulmonary infections results in biotrauma. Poor lung compliance and surfactant deficiency places the infant at risk for poor lung recruitment and alveolar instability; the sheering forces associated with the repetitive re-opening of collapsed alveoli with each breath underlie atelectotrauma. Conversely, over-distension of fragile neonatal alveoli, even for brief periods of time, stretches the alveolar walls, disrupts the underlying extracellular matrix, and incites an inflammatory cascade that results in volutrauma. The neonatal lung injury associated with biotrauma, atelectotrauma, and volutrauma, place the preterm infant at significantly increased risk of developing severe BPD.

Supporting the cardiopulmonary needs of the extremely preterm neonate while minimizing neonatal lung injury, and therefore the risk of severe BPD, can be accomplished through a comprehensive approach to neonatal mechanical ventilation. The key principles of avoiding both atelectotrauma and volutrauma are satisfied by relying on open lung ventilation. Prior to the availability of reliable neonatal volume ventilators, high frequency oscillatory ventilation (HFOV) was utilized to provide sub-physiological tidal volumes and constant distending pressure in an effort to provide lung-protective ventilation. However, metanalysis of 19 trials randomizing nearly 5,000 infants failed to show a benefit for mortality and there was only a very small, inconsistent effect to reduce BPD in survivors (30, 31). Moreover, in trials that compared modern-day conventional ventilation strategies (lower volume, higher rate) to HFOV, there was no benefit (32). Advances in neonatal ventilator microprocessor technology allowing for delivery of small tidal volumes reliably and reproducibly have led to the preferred mode of neonatal invasive ventilatory support being volume-targeted ventilation. The resulting ventilatory support allows for the benefits of a pressure-controlled mode in terms of flow while offering the lung-protective benefits of limited volume. These volume targeting/guarantee modes of ventilation utilize the lowest possible pressure to achieve a desired targeted tidal volume. Previously, during pressure-limited ventilation, rapid changes in compliance accompanying surfactant administration had the potential to translate into delivery of excessive tidal volume. The immediate response to the volutrauma that ensues is hyperventilation and air leak (pulmonary interstitial emphysema and pneumothoraces). Preclinical evidence demonstrated that even a few excessive breaths to a surfactant-deficient lung (as can happen in the delivery room or following exogenous surfactant administration) incites an enduring inflammatory cascade and lung injury (33–35). When comparing pressure-limited and volume-targeted ventilation, 20 randomized trials enrolling 1,065 infants from 1997 to 2016 demonstrated a reduction in episodes of hyperventilation, air leak [RR 0.52 (0.31–0.87)], Grade 3–4 IVH [RR 0.53 (0.37–0.77)], and BPD [RR 0.68 (0.53–0.87)] (36). Therefore, including at our institution, volume-targeted ventilation using lower tidal volumes (4–6 ml/kg), shorter inspiratory times (0.3–0.4 s), and higher rates (40–60 bpm) has become the preferred initial mode of ventilation.

To avoid both volutrauma and (as importantly) atelectotrauma, it is imperative to give judicious attention to optimizing PEEP. Maximal compliance (change in tidal volume for a given change in inspiratory pressure) occurs at optimal FRC. In the presence of inadequate PEEP, poor compliance and alveolar instability result in atelectasis. For an atelectatic distal airspace to be ventilated, the ventilator must first apply a critical opening pressure. Indeed, reaching the critical opening pressure to provide ventilation to the distal airspace utilizes a significant proportion of the inspiratory cycle and peak inspiratory pressure. Repetitive reopening of the alveolus exposes the alveolar wall to damaging shearing forces that disrupt lung structure and incite inflammation, and the expense of a significant proportion of the respiratory cycle merely to open the alveolus reduces ventilation efficacy. The impact of optimal lung inflation on oxygenation, as well as the contribution of surfactant replacement, were nicely demonstrated in 103 preterm infants (mean GA 29.4 wk) with respiratory distress syndrome requiring intubation and mechanical ventilation in the first hours of age (37). Using a step-wise titration of constant distending pressure while on high frequency ventilation, titration from closed pressure (mean 12.0 cm ± 4.0 cm H_2_O), to a fully-recruited open pressure (mean 20.5 cm ± 4.3 cm H_2_O), and back to an optimal constant distending pressure (mean 14.0 cm ± 4.0 cm H_2_O) was associated with a robust and significant reduction in supplemental oxygen needs (from mean 0.7 ± 0.27–0.24 ± 0.04). Surfactant administration was associated with a significant, further reduction in optimal distending pressure (mean 9.3 cm ± 2.6 cm H_2_O). Thus, not only does optimal recruitment protect from atelectotrauma, it also allows for a reduction in oxygen exposure.

**Figure 1:**
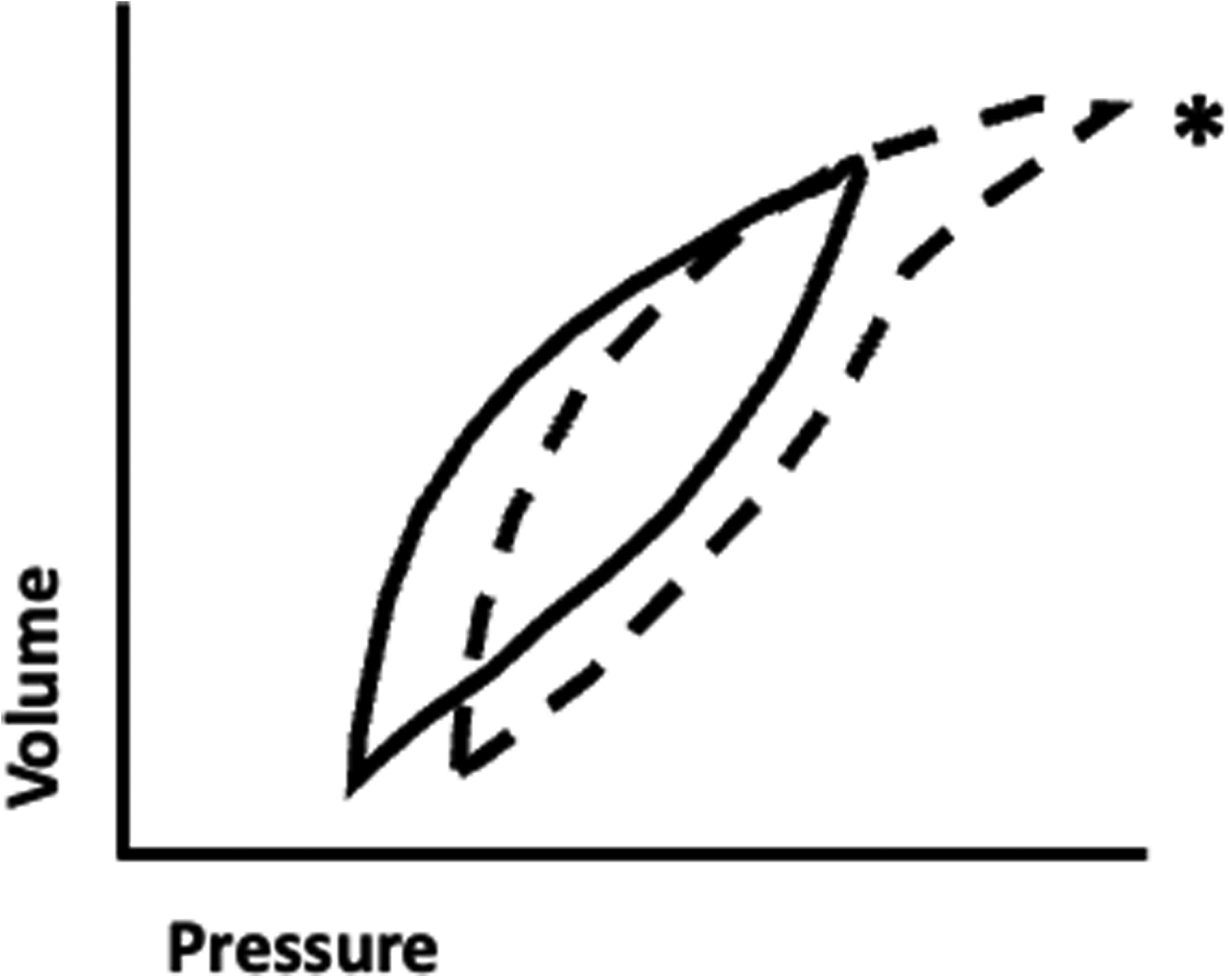
Pressure-volume loop demonstrating the phenomenon of “beaking.” A typical pressure-volume loop generated from ventilation of an optimally-expanded lung (solid line) is shown for reference. Note that in the normal condition, a relatively small change in pressure results in a significant change in volume. Excessive positive end expiratory pressure can result in ventilation of a hyperinflated lung (dashed line), and as tidal volume nears total lung capacity it requires a higher change in pressure and a higher peak pressure to attain a similar change in volume. The flattening of the upper portion of the curve is referred to as “beaking” (asterix)

Examination of the pressure-volume loop will demonstrate flattening of the initial portion with a sudden upward inflection when critical opening pressure is reached. Conversely, in the presence of excessive PEEP (either extrinsic or intrinsic), excessive pressure is required to force tidal volume into an already over-distended alveolus. Examination of the pressure-volume loop in this case will demonstrate “beaking” at the tip of the curve representing the excessive pressure required to force a relatively small amount of volume into a lung that at end-expiration is already near total lung capacity (Figure 1) (38, 39).

Clinically, the infant with atelectatic lungs will require higher fractional inspired oxygen and demonstrate oxyhemoglobin saturation (SpO2) instability due to inadequate FRC, while the infant with hyperexpanded lungs tends to require relatively lower amounts of inspired oxygen but have carbon dioxide retention and, when severe, hypotension secondary to impaired venous return. In terms of compliance, both atelectasis and over-distension require a higher change in pressure to achieve a similar change in volume when compared to an optimally-inflated lung. At optimal inflation, where atelectasis and over-distension are minimized (typically a PEEP of 4 cm–7 cm H_2_O), not only is the smallest change in pressure required to deliver a given tidal volume, both atelectotrauma and volutrauma are minimized. Therefore, during volume-target ventilation, in addition to ensuring 8–9 rib expansion on chest xray, one can titrate to optimal PEEP by monitoring and optimizing compliance.

Although avoidance and minimization of mechanical ventilation is ideal, for a subset of infants mechanical ventilation will be prolonged (40). In infants remaining on volume-targeted ventilation beyond the first week of age, it is imperative they be monitored for tidal volume evolution. Although initial tidal volumes of 4–5 ml/kg are sufficient to support infants with early RDS (41, 42), several lines of evidence in infants requiring mechanical ventilation for the first month support the need to modestly increase tidal volumes. In a retrospective observational study of 26 infants with birthweights <800 g cared for on volume-targeted ventilation over the first three weeks of age, exhaled tidal volumes associated with target carbon dioxide (PCO2) levels were examined for evolution. Over the first 3 weeks of age, mean exhaled tidal volumes increased significantly from a mean of 5.15 ml/kg (day 1–2) to 6.07 ml/kg (day 18–21) (41). In a similar observational study of 18 infants with median GA of 25 weeks' who were ventilated for the first 28 days of age, despite carbon dioxide levels increasing significantly (from a mean PCO2 42 mmHg to 60 mmHg), mean exhaled tidal volumes steadily rose from 5.4 to 7.2 ml/kg. There was a corresponding increase in mean minute ventilation (263–368 ml/kg/min) and a modest increase in peak inspiratory pressures (18.1–22.4 cm H_2_O) (43). The authors speculated that to maintain relative normocapnia, the increase in minute ventilation and tidal volume were required to compensate for an expansion of the anatomical dead space (*via* both distension of the upper airways as well as alveolar airspace dilation).

Anatomical dead space is relatively increased in extremely preterm infants and expands with mechanical ventilation. In an observational study of 45 premature infants (median 25 weeks' GA) ventilated for a median of 8 days compared to 11 term infants, despite similar tidal volumes (5.6 and 5.3 ml/kg, respectively) the preterm infants had significantly larger anatomical (3.7 vs. 2.4 ml/kg) and alveolar component (0.3 vs. 0.1 ml/kg) of dead space (44). The increase in dead space was accompanied by higher respiratory rates (median 71 vs. 55 breaths/min), presumably to maintain adequate alveolar ventilation. Notably, anatomical dead space in relation to body weight (ml/kg) was inversely proportional to birthweight and gestational age. Thus, likely owing to a relatively fixed endotracheal tube volume, smaller and more immature infants have a relatively higher proportion of tidal volume occupied by the anatomical dead space. Anatomical dead space is directly proportional to days of mechanical ventilation, and it is significantly higher in infants that go on to develop BPD (45).

**Figure 2:**
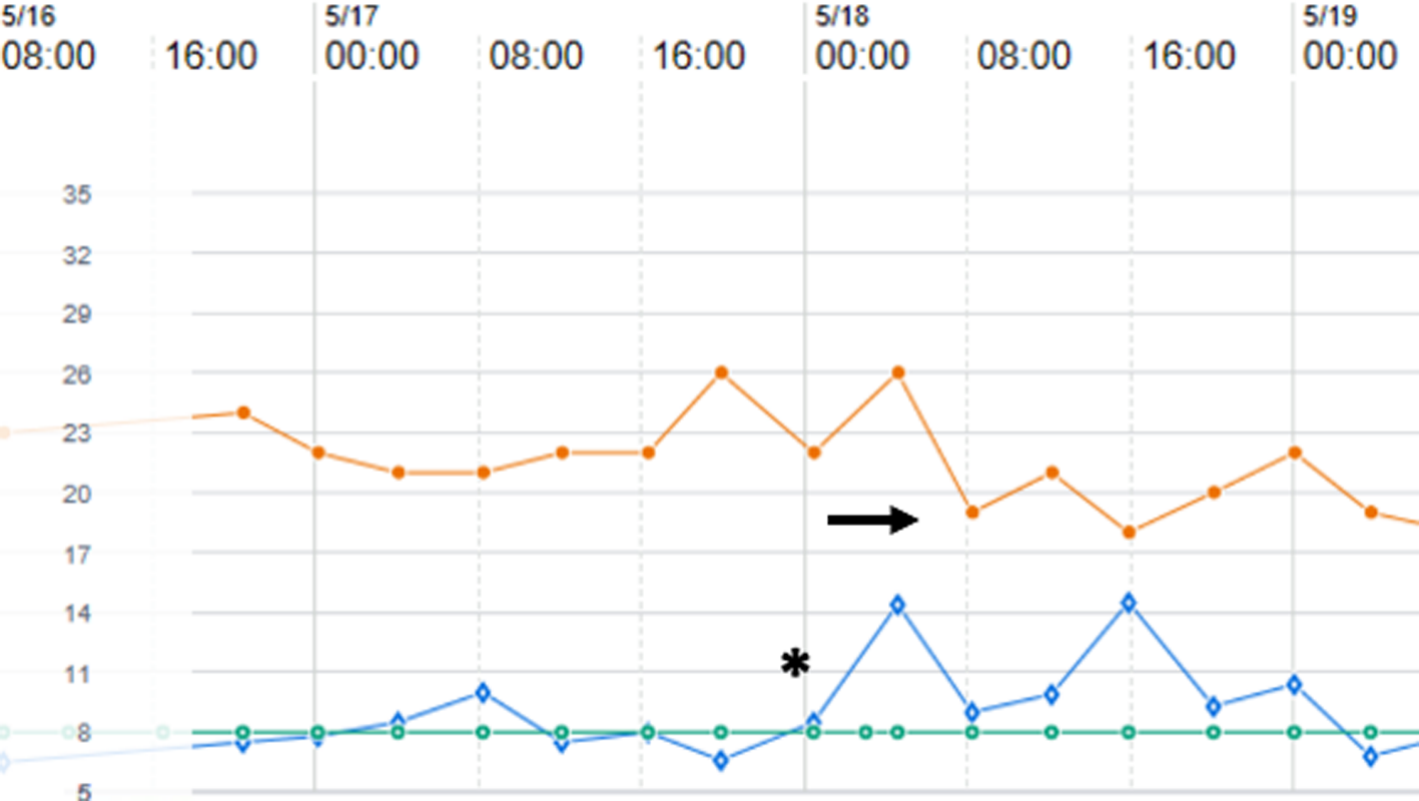
Ventilator data flowsheet encompassing 18–21 days of age for a former 27 week, 1,160 gram premature infant with evolving chronic lung disease and supported on pressure-regulated volume control ventilation. As exhaled tidal volume (open diamonds) exceeds the set tidal volume (open circles) (asterix), note that the ventilator compensates by lowering peak inspiratory pressure (closed circles) (arrow).

When ventilating a preterm infant beyond the first week of age, it is imperative to account for growth of anatomical dead space and its contribution to tidal volume evolution. Ventilator algorithms that allow for volume-targeted ventilation adjust the peak inspiratory pressure based on exhaled (measured) tidal volume. Over the first 3–4 weeks of age, infants that have continuously required mechanical ventilation will demand more tidal volume to account for an increase in anatomical dead space. Unless set tidal volume is increased, as infants inspire additional tidal volume to compensate for dead space, the ventilator will attempt to maintain the set tidal volume by reducing peak inspiratory pressure. The result is the infant must assume more of the work of breathing. In an observational study of 18 ventilator-dependent infants (24–30 weeks’ GA) studied at a median of 18 days of age (range 7–60 days), as tidal volume was advanced from baseline (5.8 ml/kg) to 7 ml/kg, there was significantly lower work of breathing (46). Additionally, mean respiratory rates fell as tidal volume was increased (from 54 breaths/min at baseline to a 44 breaths/min at 7 ml/kg). At all tidal volumes, minute ventilation was similar but peak inspiratory pressures increased significantly (from 19.7 cm to 24.3 cm H_2_O). Similar results were noted by the same authors in a prior trial (47). Thus, over the first 3–4 weeks of age, it is prudent to compare exhaled to set tidal volumes. As exhaled tidal volumes exceed set tidal volumes, there will be an accompanying decrease in peak inspiratory pressures (Figure 2). The decrease in inspiratory pressures is often erroneously viewed as improved compliance, when in actuality it is a product of ventilator compensation. If mechanical ventilation is still required, an increase in set tidal volume (usually to 6–7 ml/kg) to match the patient's effort will often result in improved ventilation and patient comfort.

## Chronic mechanical ventilation

Although the overwhelming majority of premature infants will eventually wean from positive pressure ventilation, some will need chronic respiratory support. There is no clear timing when providers should transition to chronic ventilator strategies, and there is wide variation based on center (48). However, once it has been determined that an infant with BPD will be treated with chronic mechanical ventilation, the ventilation strategy should shift. While there should be continued efforts to minimize lung injury as much as possible, the primary focus transitions to providing optimal respiratory support for patient comfort, growth and development, and gas exchange, which appears to improve in neonates with BPD following placement of a tracheostomy tube and chronic mechanical ventilation (49).

**Figure 3:**
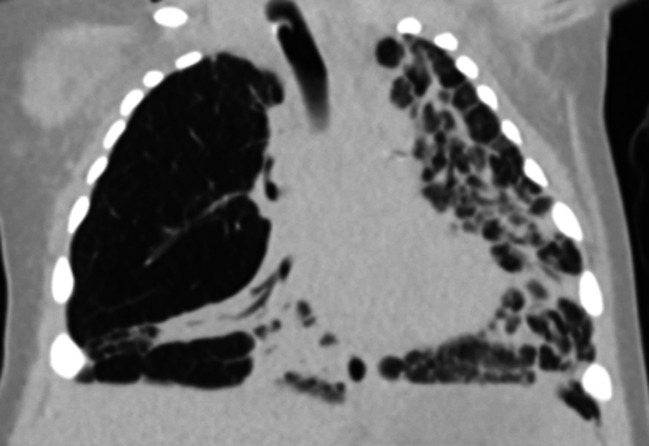
Chest computed tomography of an 8 month old former 26 week premature infant with severe BPD demonstrating dramatic pulmonary heterogeneity with alveolar simplification of the right upper lobe and diffuse fibrosis of the left lung. The patient is also severely hyperinflated with flattened diaphragms.

Currently, there is an extreme paucity of data comparing different chronic ventilator strategies in established severe BPD; consequently, a physiologic approach to mechanical ventilation must be considered. Neonates with severe BPD have extensive pulmonary heterogeneity with areas of alveolar simplification and air-trapping as well as scarring and fibrosis (Figure 3) resulting in high respiratory system resistance and low respiratory system compliance (50, 51); further, small and large airway disease are also frequently encountered (52, 53). As a result of these complex interactions, neonates with severe BPD may have obstructive, mixed obstructive and restrictive, or restrictive respiratory disease, with 90% developing at least some degree of obstruction (53). Thus, ventilator strategies are typically designed for obstructive respiratory disease with severe pulmonary heterogeneity.

### Trigger

**Figure 4:**
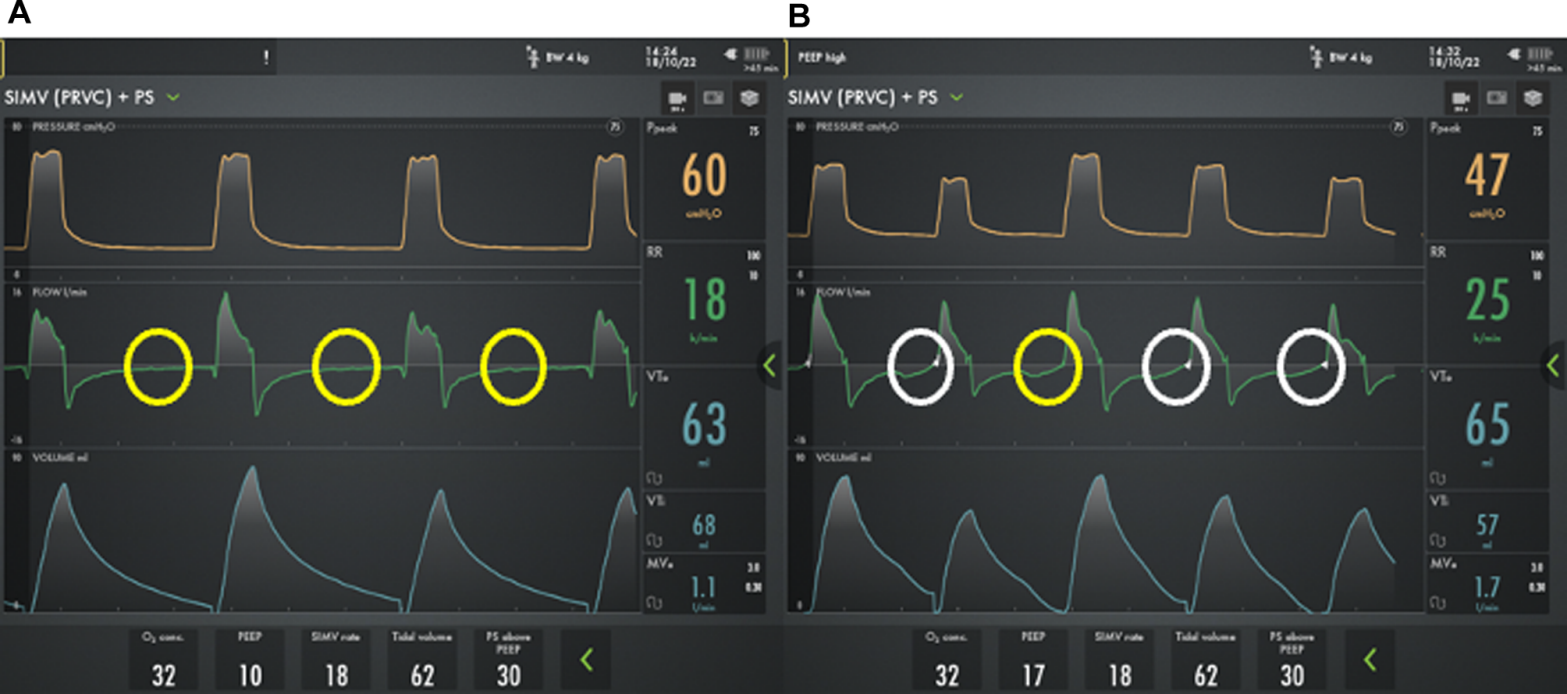
Ventilator waveforms from a 2 month old former 24 week premature infant with severe bronchopulmonary dysplasia using two different PEEP strategies. On physical exam, the child has a respiratory rate of 26 breaths/min. **A**) However, using a PEEP 10 cm H_2_O, he has multiple failed triggers (yellow circles) and does not appear to breathe above the mandated rate. **B**) By increasing the PEEP to 17 cm H_2_O there is improved synchronization with the ability to trigger his spontaneous breaths, though the trigger is delayed (white circles) and one patient effort fails to trigger a breath (yellow circles).

Most providers will rely on a combination of time and flow triggers for chronic mechanical ventilation. A time trigger will initiate a breath based on the set respiratory rate regardless of patient effort. However, flow triggered breaths rely on the patient to generate inspiratory flow to initiate the breath. Obstructive lung disease can lead to an increase in intrinsic PEEP (PEEPi), and the baby will then have to overcome the PEEPi prior to generating the airflow needed to trigger a breath. As a result, patients with established severe BPD may have delayed triggers or failed triggers (Figure 4A). In one report, a majority patients with severe BPD treated with chronic mechanical ventilation experienced failed triggers, with nearly 15% of breaths resulting in a wasted effort. The inability to trigger, results in patient ventilator dysnchronization and patient discomfort. This may manifest with agitation, hypoxemic episodes, poor ventilation, and increased need for sedation (54).

If a patient is having difficulty triggering a breath, the flow trigger can be made as sensitive as possible without generating autocycling where the ventilator initiates a spontaneous breath without a patient effort. Further, increasing or decreasing PEEP to match PEEPi can improve triggering and should be considered (Figure 4B) (54). If optimization of respiratory mechanics does not permit adequate patient-ventilator interaction, NAVA can be used. NAVA relies on diaphragmatic activity for synchronization rather than the generation of airflow and can improve synchronization and gas exchange and reduce respiratory work and the need for sedation in patients with severe BPD (55–57). Despite the potential benefits, NAVA is only implemented in a minority of centers that care for patients with severe BPD and is not compatible with home ventilators.

### PEEP

Management of PEEP can be challenging in patients with severe BPD but is critical to prevent atelectasis and maintain FRC. Currently there are no large studies to define the optimum PEEP for patients with severe BPD requiring chronic ventilation. Titration of PEEP to match PEEPi can improve patient triggering and patient comfort as described above. PEEP can also be titrated during bronchoscopy to maintain airway lumen patency in patients with severe BPD and tracheobronchomalacia (58), which is quite common and associated with increased respiratory morbidity in neonates with severe BPD (52, 59). For these children with dynamic central airway collapse, PEEP can improve respiratory mechanics and increase expiratory flow rates (60, 61). Similarly, the titration of PEEP can help minimize dynamic collapse of smaller airways, and fairly high PEEP (>15 cm H_2_O) may be necessary in patients with more severe disease (62). Paradoxically, higher PEEP may result in reduce rather than increase hyperinflation by preventing dynamic airway collapse. However, excessive PEEP can worsen hyperinflation and decrease respiratory system compliance as described above. Thus, PEEP must be adjusted to match the physiology of each patient.

To objectively titrate PEEP, esophageal pressure can be measured as a surrogate for pleural pressure and PEEP adjusted to match pleural pressure. In cases where esophageal manometry is not feasible or not available, measuring static respiratory mechanics using an expiratory hold can identify the severity of PEEPi, and an inspiratory hold at increasing leveles of PEEP, often called a “PEEP grid,” can determine the PEEP that optimizes respiratory system compliance and resistance, which is generally ideal for chronic respiratory support (62, 63). Forced oscillation technique (FOT) can also be used to objectively measure respiratory system resistance and reactance in neonate with BPD and is becoming increasingly available; thus, FOT could prove useful when titrating PEEP as well as measuring response to other interventions (64).

### Tidal volume

When considering the tidal volume needed for chronic ventilatory support in children with severe BPD, it is critical to understand that the lung parenchyma is quite heterogenous, with some units having long time constants and others that have shorter time constants, and these patients have a significant increase in dead-space. As a result, it is typically necessary to implement a strategy of longer inspiratory times and lower mandatory respiratory rates to ensure all respiratory units are adequately ventilated and allowed to empty. Because emptying requires a prolonged expiratory time (and, therefore, fewer breaths per minute), maintenance of adequate minute ventilation requires a larger tidal volume strategy (65). Such a strategy is in stark contrast to that required for acute respiratory distress syndrome, which typically relies on low tidal volume, short inspiratory times, and high mandatory rates to minimize lung injury. While there is no universally agreed upon tidal volume, support should be titrated to the individual patient's need; most authors suggest a tidal volume of 8–12 ml/kg for children with severe BPD and chronic respiratory failure, and some advocate the use of up to 15 ml/kg (62, 63, 65, 66).

**Figure 5:**
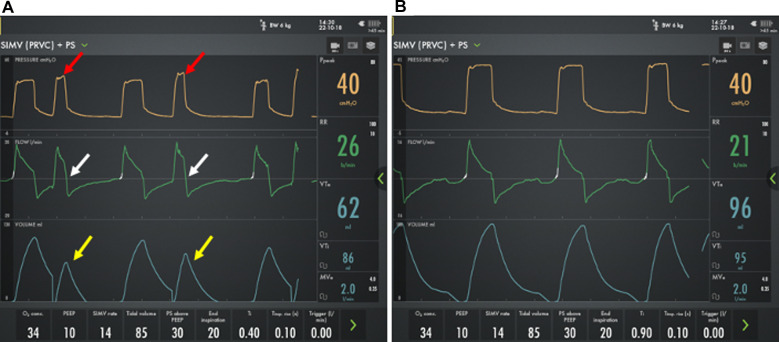
Ventilator waveforms from an 8 month old former 24 week premature infant with severe bronchopulmonary dysplasia using two different inspiratory time strategies. (**A**) Using a short (0.4 s) inspiratory time, there is incomplete filling (white arrows) with reduced tidal volume (yellow arrows), increased respiratory rate, and increased peak pressures (red arrows) compared with (**B**) a long (0.9 s) inspiratory time.

Because of the need for large mandatory breaths and significant heterogenous lung disease, inspiratory times of 0.6 s or more are often needed to ensure adequate filling of respiratory units, especially those with long time constant. Generally, the inspiratory time should be titrated to allow nearly complete filling as can be identified by the flow-time curve approaching zero inspiratory flow based on ventilator graphics (Figure 5) combined with chest auscultation. Failure to provide adequate inspiratory time will result incomplete filling and dead-space ventilation. Similarly, pressure supported breath should be supported with a low flow cycle sensitivity (typically 20%–30%) and utilize pressures that result in tidal volumes similar to those achieved during a mandatory breath (62, 63, 65, 66).

### Mandated respiratory rate

While ensuring adequate filling of respiratory units with differing time-constants using a long inspiratory time strategy, it is also critical to ensure that children with BPD have adequate time to exhale. Because children with BPD typically have obstructive respiratory disease, long expiratory times are needed to prevent incomplete exhalation and dynamic air-trapping. Thus, low mandatory respiratory rates (<20 breaths per minute) should be implemented. This combination of a low rate, large breath, long inspiratory time strategy, will provide adequate minute ventilation and maximize gas distribution to the entire lung despite the pulmonary heterogeneity, thus maintaining reasonable gas exchange.

### Gas exchange

There are currently no trials that define the optimal SpO2 nor PCO2 for infants with established BPD who need chronic ventilation. Sustained hypoxemia with SpO2 below 92% during sleep time has been correlated with growth failure and should be avoided (67, 68). while targeting SpO2_ _> 93% may reduce the need for rehospitalization following the initial discharge (69). Further, permissive hypercapnia is generally tolerated in this patient population due to the increased dead-space; however, given the risk of pulmonary hypertension, targeting PCO2 less than 60 mm Hg and a neutral pH is likely prudent (70). Because of the limited data surrounding optimal gas exchange for chronic ventilation in children with BPD, it is more important to utilize a strategy that allows adequate growth and respiratory comfort and optimizes development and tolerance of care.

### Transition to home ventilators

Once infants are stable on chronic ventilator settings and large enough (>5 kg for most ventilators), transition to a home ventilator can be considered. While reports do exist describing methods for transition to home ventilators (71), practice patterns vary considerably by center. In general, most providers will attempt to transition to the home ventilator on settings consistent with the hospital ventilator. In some situations, it may not be possible to achieve identical settings. Trigger sensitivity is less for home ventilators, which can lead delayed or failed triggers during the transition. Further, hospital ventilators may allow a longer inspiratory time than is feasible on a home ventilator, particularly with smaller tidal volumes, which can be problematic in children who need long inspiratory times to ensure recruitment of regions with long time constants (62).

For many home ventilators, it may also be necessary to transition from an active, double-limb circuit to a passive, single-limb circuit. Passive, single-limb circuits can be particularly challenging as the ventilators rely on algorithms rather than direct measurement of tidal volume and typically underestimate the volume delivered to the patient. As a result, minute ventilation may be reduced resulting in hypercapnia and increased respiratory effort. Additionally, passive, single-limb circuits require inspiration and exhalation *via* the same tubing and rely on exhalation through a fixed leak in the circuit such as a Whisper Swivel (Respironics). As a result, there is risk of rebreathing exhaled gas. To prevent this, the ventilator delivers a continuous flow of gas to washout the dead space; however, if insufficient flow is delivered, the patient may develop hypercapnia. Active exhalation can also be used to avoid this issue (72). If available, a double-limb circuit can be used to avoid the challenges of a single-limb circuit; unfortunately some home ventilators do not provide this option.

These differences among many others may results in difficulty tolerating the home ventilator. If a child fails the initial transition to a home ventilator, most centers will wait one to two weeks prior to attempting the transition again. Once the child has successfully transitioned to a home ventilator, preparation for discharge home should commence.

### Transition to home

While transition to home is often an exciting time for families, many care givers report significant depression and anxiety surrounding discharge. Caregivers also experience reduced quality of life and increased fatigue related to the burden of care for technology dependent children at home, though this can be mitigated if home nursing support is available (73–75). Fatigue is of particular importance as in home mortality in this population exceeds 15%, and many of the events resulting in the patient's demise are preventable or treatable e.g., mucus plugs or accidental decannulation rather than progression of the underlying lung disease (76–79). Because of the risks, most programs provide extensive training for caregivers of technology dependent children that center on management of the tracheostomy, ventilator, and all other equipment that will be necessary to meet the child's needs at home. Caregivers should also be trained in cardiopulmonary resuscitation and patient transfers. These skills can be demonstrated with a combination of simulation training and independent stays prior to hospital discharge (80–86).

### Weaning chronic mechanical ventilation

Children with BPD who are discharged with home mechanical ventilation are expected to gradually wean ventilatory support over a period of months to years, and nearly all children are liberated from mechanical ventilation by 5 years of age (79). There is no specific, validated protocol for weaning mechanical ventilation in the ambulatory setting, and practices vary by both provider and institution (63). Typically, patients are allowed progressively increased periods off mechanical ventilation during the day. Once off support during the day, nocturnal support is then discontinued. This may be done at home, during a short hospital admission, or with the aid of polysomnography (87, 88).

### Weaning non-invasive support

As with weaning of mechanical ventilation, there is precious little data for the optimal strategies to wean non-invasive support following hospital discharge. In one small case series, 12/17 (71%) patients with BPD discharged using HFNC were able to successfully wean to room air after an average of about 6 months. However, 4/17 (24%) of these patients died prior to weaning from support, which is much higher than demonstrated in other cohorts of patients with severe BPD (89, 90). Because of the high rates of mortality following discharge, home HFNC should be considered with extreme caution in neonates with BPD. The experience with non-invasive positive pressure is limited to case reports (91); thus, the safety and efficacy of home non-invasive positive pressure remains largely unknown.

## Conclusion

Ventilator strategies for children born premature evolve as the disease process progresses. While there is currently a wealth of information highlighting the use lung protective strategies with non-invasive positive pressure ventilation or invasive ventilation with small tidal volumes and high mandatory rates during the earliest phase of disease, there is a dearth of data about the timing of transition to chronic respiratory support and the optimal chronic ventilatory strategies. Ultimately, children will gradually wean from support, typically by school-age. Prospective trials that establish optimal ventilator strategies for children with severe established BPD are desperately needed, and the need for such studies continues to grow as the limit of viability is decreased and more children will need chronic mechanical ventilation.
